# Novel Toscana Virus Reverse Genetics System Establishes NSs as an Antagonist of Type I Interferon Responses

**DOI:** 10.3390/v12040400

**Published:** 2020-04-04

**Authors:** Franziska Woelfl, Psylvia Léger, Nadia Oreshkova, Felix Pahmeier, Stefan Windhaber, Jana Koch, Megan Stanifer, Gleyder Roman Sosa, Zina M. Uckeley, Felix A. Rey, Steeve Boulant, Jeroen Kortekaas, Paul J. Wichgers Schreur, Pierre-Yves Lozach

**Affiliations:** 1 CellNetworks Cluster of Excellence, University Hospital Heidelberg, 69120 Heidelberg, Germany; Woelfl@stud.uni-heidelberg.de (F.W.); pleger.heidelberg@gmail.com (P.L.); felix.pahmeier@googlemail.com (F.P.); stefan_windhaber@arcor.de (S.W.); Jana.Koch@med.uni-heidelberg.de (J.K.); Zina.Uckeley@med.uni-heidelberg.de (Z.M.U.); 2 Center for Integrative Infectious Diseases Research (CIID), Virology, University Hospital Heidelberg, 69120 Heidelberg, Germany; s.boulant@Dkfz-Heidelberg.de; 3 Wageningen Bioveterinary Research, Department of Virology, 8221 RA Lelystad, The Netherlands; nadia.oreshkova@wur.nl (N.O.); jeroen.kortekaas@wur.nl (J.K.); 4 Center for Integrative Infectious Diseases Research (CIID), Molecular Virology, University Hospital Heidelberg, 69120 Heidelberg, Germany; m.stanifer@dkfz-heidelberg.de; 5 Structural Virology Unit, Pasteur Institute, 75015 Paris, France; gromansosa@vet.k-state.edu (G.R.S.); felix.rey@pasteur.fr (F.A.R.); 6 Laboratory of Virology, Wageningen University, 6708 PB Wageningen, The Netherlands; 7 INRAE, EPHE, Viral Infections and Comparative Pathology (IVPC), University Claude Bernard Lyon1, University of Lyon, UMR754, 69007 Lyon, France

**Keywords:** Arbovirus, *Bunyavirales*, interferon, neglected diseases, *Phenuiviridae*, *Phlebovirus*, reverse genetics, sand fly fever, Toscana virus

## Abstract

The sand fly-borne Toscana virus (TOSV) is the major cause of human meningoencephalitis in the Mediterranean basin during the summer season. In this work, we have developed a T7 RNA polymerase-driven reverse genetics system to recover infectious particles of a lineage B strain of TOSV. The viral protein pattern and growth properties of the rescued virus (rTOSV) were found to be similar to those of the corresponding wild-type (wt) virus. Using this system, we genetically engineered a TOSV mutant lacking expression of the non-structural protein NSs (rTOSVɸNSs). Unlike rTOSV and the wt virus, rTOSVɸNSs was unable to (i) suppress interferon (IFN)-b messenger RNA induction; and (ii) grow efficiently in cells producing IFN-b. Together, our results highlight the importance of NSs for TOSV in evading the IFN response and provide a comprehensive toolbox to investigate the TOSV life cycle in mammalian and insect host cells, including several novel polyclonal antibodies.

## 1. Introduction

Toscana virus (TOSV) is a sand fly-borne pathogen of the genus *Phlebovirus* (order *Bunyavirales*, family *Phenuiviridae*) that is responsible for neuro-invasive infections in humans causing meningitis and encephalitis [1,2]. The virus was first isolated in 1971 from *Phlebotomus perniciosus* and *Phlebotomus perfiliewi* sand flies in the Tuscany region of central Italy [2,3]. TOSV is re-emerging in the Mediterranean basin, as shown by an increasing number of outbreaks and reported cases in southern Europe, which includes France, Spain, and Italy, during the last decade [1,4]. However, to date, no vaccines or treatments are available for human use.

Similar to other phleboviruses, TOSV particles are enveloped and contain a tri-segmented single-stranded RNA genome, which is exclusively replicated in the cytosol of infected cells [5,6]. The small genomic segment (S) encodes the nucleoprotein N and the non-structural protein NSs in an ambisense orientation [7]. Specifically, the N protein is translated from subgenomic messenger RNAs (mRNAs) directly transcribed from the viral genomic RNA (vRNA), whereas the NSs protein is translated from an mRNA transcribed from the antigenomic, replicative-intermediate RNA [8,9]. The medium segment (M) codes the non-structural protein NSm and the two envelope glycoproteins G_N_ and G_C_, while the large segment (L) encodes the RNA-dependent RNA polymerase L, all in genomic sense orientation. Inside the viral particles, the viral genome is present as a ribonucleoprotein (RNP) complex bound to a polymerase L molecule. On viral particles, G_N_ and G_C_ are organized in a surface lattice. The two envelope glycoproteins are responsible for virus attachment to target cells, and for entry into the cytosol upon acid-activation membrane fusion in endosomal vesicles [5,6].

The life cycle of TOSV remains largely uncharacterized at a molecular and cellular level. Heparan sulfate and the human C-type lectins DC-SIGN and L-SIGN were shown to be involved in the attachment of viral particles to the cell surface [10,11,12,13]. Following the release of the genomic RNPs into the cytosol, the virus makes use of cap-snatching to initiate viral mRNA synthesis [14]. GBF1, a guanine nucleotide exchange factor resident to the Golgi, was recently demonstrated to contribute to viral replication, assembly, and egress [15], although the mechanistic details are yet to be elucidated. Other phleboviruses have been shown to counteract type I interferon (IFN) responses [16]. The TOSV NSs protein was proven to act as an IFN antagonist when expressed from transfected plasmids, or from the genome of Rift Valley fever virus (RVFV), another member of the genus *Phlebovirus* [17,18]. Using similar approaches, TOSV NSs was shown to have an E3 ubiquitin ligase activity that promotes RIG-I degradation [19].

In this study, we describe a reverse genetics system to recover the H4906 strain (lineage B) of TOSV (rTOSV) from cDNAs. We also report the generation of polyclonal antibodies against TOSV NSs, N, G_N_, and G_C_. Using these tools, we rescued and characterized a mutant virus lacking NSs expression (rTOSVɸNSs). Our results confirm that the TOSV NSs protein, authentically expressed from the viral genome, functions as an antagonist of type I IFN responses.

## 2. Materials and Methods

### 2.1. Cells and Viruses

All products used for cell culture were obtained from Thermo Fisher Scientific and Sigma-Aldrich. Baby hamster kidney cells (BHK-21) were grown in Glasgow’s minimal essential medium (GMEM), supplemented with 10% tryptose phosphate broth, 5% fetal bovine serum (FBS), 1% GlutaMAX, 100 units/mL penicillin, and 100 µg/mL streptomycin. BHK-21 cells stably expressing T7 RNA polymerase (BHK/T7-9 cells) were kindly provided by N. Ito (Gifu, Japan) [20]. The selection of BHK-21 cells that express T7 polymerase was achieved every two passages in the presence of hygromycin B. Human epithelial lung cells (A549) were grown in Dulbecco’s modified eagle medium, supplemented with 10% FBS, 1% non-essential amino acids, 100 units/mL penicillin, and 100 µg/mL streptomycin. Monkey epithelial kidney cells (Vero) were grown in the same medium as the A549 cells, but lacking non-essential amino acids. TOSV strain H4906 (lineage B) and Germiston virus (GERV) have been described previously (Genbank accession numbers KU922126.1, KU922125.1, and KU922127.1, and [21], respectively).

### 2.2. Antibodies and Reagents

Polyclonal antibodies against all TOSV (T1) and GERV (GR1) proteins, as well as against the TOSV envelope glycoproteins G_N_ (T2) and G_C_ (T3), were made by the Antibody Unit of the Genomics and Proteomics Core Facility of the German Cancer Research Center, Heidelberg (Table 1). Briefly, viruses were produced and purified, as described in Section 2.5. Soluble ectodomains of G_N_ and G_C_ were stably expressed in drosophila S2 cells and purified according to a standard procedure [22]. Using these protocols, the protein purity reached over 90%. Antisera were prepared in guinea pigs by injecting 100 µg of Triton X-100-inactivated purified viruses or soluble ectodomains of G_N_ and G_C_ in Freund’s complete adjuvant. The priming was followed by three booster injections of 100 µg at 4-week intervals, the first one in Freund’s incomplete adjuvant, and the others in phosphate-buffered saline (PBS). Animals were bled before the first immunization (control pre-immune serum) and 9 days after the last injection. The purified rabbit polyclonal antibodies against the TOSV nucleoprotein N (T4) and non-structural protein NSs (T5) were developed by GenScript (Piscataway, The Netherlands). Rabbits were immunized with either a peptide corresponding to the C terminal 14 amino acid residues of NSs or the full-length N protein with a C-terminal His-tag. The mouse immune ascitic fluid against all TOSV structural proteins was a generous gift from R.B. Tesh (University of Texas, Galveston, Texas, USA).

### 2.3. Plasmids

Plasmids were constructed by GenScript. Briefly, the cDNAs encoding the full-length S, M, and L segments of TOSV, flanked by an upstream T7 polymerase promoter sequence and a downstream hepatitis delta virus (HdV) ribozyme sequence (Figure S1), were synthesized. The gene synthesis products were subjected to blunt-end ligation into EcoRV-linearized pUC57 plasmid, or alternatively, were cloned into the pCC1 plasmid, using the CloneEZ PCR Cloning Kit (Genscript), resulting in pUC57-S, pCC1-M, and pUC57-L. In addition, a pUC57-SɸNSs was created in which the S segment encodes NSs mRNA, with the 18 first AUG codons replaced by UAG stop codons (Figure S2).

### 2.4. Rescue of Viruses from Plasmid DNAs

BHK-21 cells expressing T7 polymerase were seeded in 6-well plates (2.5 × 10^5^ cells per well). The following day, rTOSV was rescued from cells by transfection with 1 µg each of the plasmids pUC57-S, pCC1-M, and pUC57-L. Alternatively, the recovery of rTOSVɸNSs was achieved following the same method but using the plasmid pUC57-SɸNSs instead of the pUC57-S. Transfection was performed with Lipofectamine 2000 (Thermo Fisher Scientific), using a ratio of 1 µL Lipofectamine 2000 to 1 µg of plasmids in 400 µL of complete GMEM without antibiotics. Supernatants were replaced by a fresh culture medium containing antibiotics and 2% serum 4 h post-transfection. Five days post-transfection, supernatants were harvested, clarified, and titrated. The rescued viruses were then passaged for a minimum of five times, once in BHK-21 cells and four successive times in Vero cells.

### 2.5. Virus Production, Purification, and Titration by Plaque-Forming Unit Assay

Virus production and purification were performed through sucrose gradient, as recently described [23]. With this method, the purity of viral proteins in virus stocks was typically superior to 90%. For titration, confluent monolayers of BHK-21 or Vero cells were infected with 10-fold dilutions of virus in FBS-free medium and then grown in the presence of complete medium containing 1% agarose to prevent virus spread. Plaques were stained by crystal violet five days post-infection. The multiplicity of infection (MOI) is given according to the titer determined on BHK-21 or Vero cells, which was similar for virus stocks on the both cell types.

### 2.6. Western Blot Analysis

A549 cells were infected at a MOI of 2 for 24 h and lysed with PBS containing 0.1% Triton X-100 (Merck Millipore), according to a standard procedure [24]. Proteins obtained from infected A549 cell lysates or from purified virus stocks were diluted in LDS sample buffer (Thermo Fisher Scientific), and analyzed by SDS-PAGE (Nu-PAGE Novex 10% Bis-Tris gels; Thermo Fisher Scientific). Proteins were subsequently transferred to polyvinylidene difluoride membranes (iBlot transfer stacks; Thermo Fisher Scientific). The membranes were first blocked with 5% milk and then incubated with primary polyclonal rabbit or guinea pig antibodies (1:1,000), all diluted in Tris-buffered saline containing 0.1% Tween and 5% milk. After extensive washing, the membranes were incubated with the corresponding anti-species conjugated to horseradish peroxidase (1:10,000; Santa Cruz). Proteins were detected by enhanced chemiluminescence reagents (ECL; Thermo Fisher Scientific), and an iNTAS ECL Chemostar analyzer. Alternatively, viral proteins were analyzed by SDS-PAGE and Coomassie blue staining, as recently described [25].

### 2.7. Infection Assay

A549 cells were infected with virus at the indicated MOIs in an FBS-free medium at 37 °C for 1 h. *Inocula* were subsequently replaced by a complete culture medium and the infected cells were incubated up to 48 h before fixation. Infection was monitored either by confocal microscopy or flow cytometry. For fluorescence microscopy analysis, infected cells were fixed with 4% paraformaldehyde, permeabilized with PBS containing 0.1% Triton X-100, and blocked using 5% bovine serum albumin in PBS for 30 min. Samples were then incubated with the anti-TOSV mouse antibody (1:1,000) at room temperature (RT) for 1 h, washed, and exposed to an Alexa Fluor (AF) 568-conjugated secondary anti-mouse antibody (1:800; Thermo Fisher Scientific, Waltham, USA) at RT for 1 h. Nuclei were subsequently stained with Hoechst 33258 (0.5 µg/mL; Thermo Fisher Scientific). Infected cells were imaged with a Leica TCS SP2 spinning disc microscope. The flow cytometry-based infection assay has been described previously [26]. Briefly, after fixation and permeabilization in the presence of 0.1% saponin, infected cells were incubated with the anti-TOSV mouse antibody (1:4,000) at RT for 1 h, washed, and incubated with AF488-conjugated secondary anti-mouse antibodies (1:500; Thermo Fisher Scientific) at RT for another 1 h. Infection was quantified with a FACSCelesta cytometer (Becton Dickinson, Franklin Lakes, USA) and FlowJo software (TreeStar, Franklin Lakes, USA).

### 2.8. IFN mRNA Quantification Assay

RNA was isolated from infected cells using the NucleoSpin RNA extraction kit (Macherey-Nagel, Düren, Germany), as per the manufacturer’s instructions. The cDNA was obtained using iScript reverse transcriptase (BioRad, Hercules, USA) from 250 ng of total RNA, according to the supplier’s instructions. Real-time quantitative reverse transcription PCR (qRT-PCR) was performed using SsoAdvanced SYBR green (BioRad) and primers shown in Table 2, following the manufacturer’s instructions. HPRT1 was used for normalization.

### 2.9. Statistical Analysis

The data shown are representative of at least three independent experiments. Values are given as the means of triplicates ± standard deviations (SD), except for the titration of virus passages. Graph plotting of numerical values, as well as the statistics, were achieved with GraphPad Prism. Statistical methods and parameters are indicated in the figure legends when applicable. P-values are shown when statistical differences are significant.

## 3. Results

### 3.1. Recovery of TOSV H4906 from cDNA

**Figure 1:**
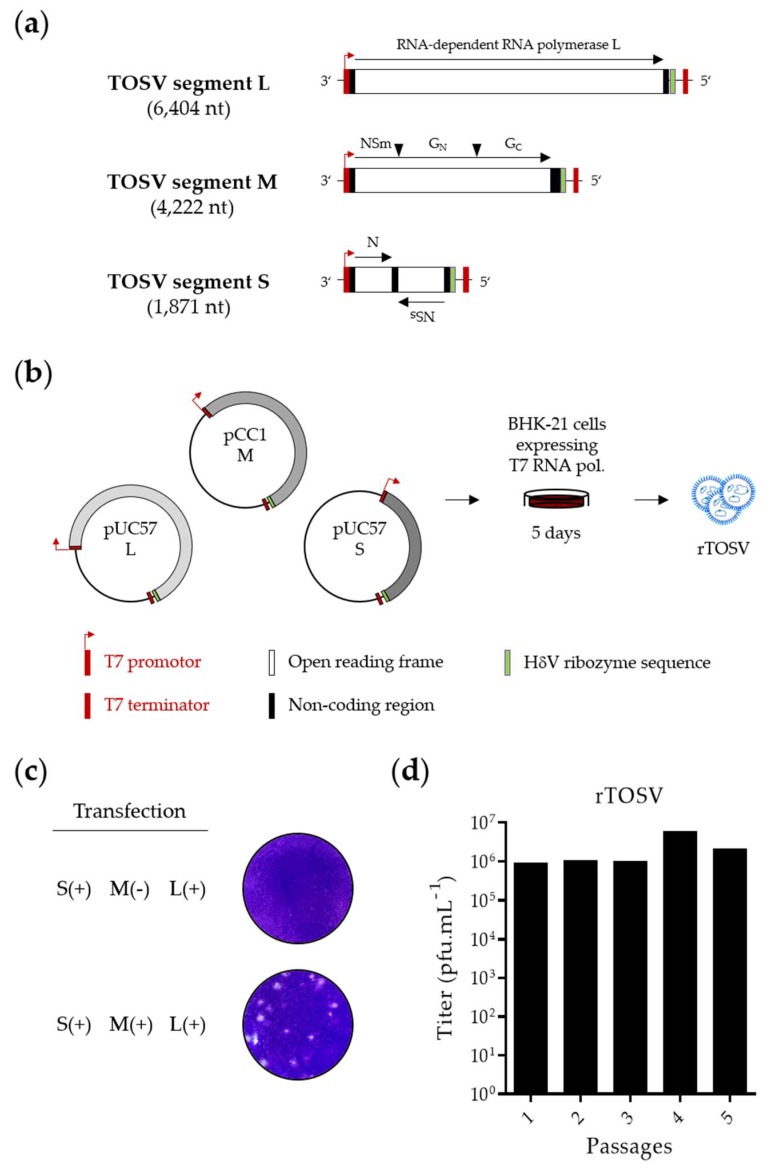
Rescue of Toscana virus (TOSV) from T7-driven plasmid DNAs. (**a**) The three negative-sense RNA genomic segments of TOSV strain H4906 (lineage B) were cloned between a T7 promoter and T7 terminator into the plasmids pUC57 (S and L) and pCC1 (M). A hepatitis delta virus (HdV) ribozyme sequence was introduced to generate authentic 3′ terminal ends. The black arrowheads mark cleavage sites in the M polyprotein precursor; (**b**) Schematic representation of the T7-driven TOSV rescue system; (**c**) Plaque-forming unit assays used for titration on BHK-21 cells of TOSV rescued from BHK-21 cells (rTOSV). After five days of incubation at 37 °C, plaques were visualized by crystal violet staining; (**d**) Titer of rTOSV after one passage in BHK-21 cells and four successive passages in Vero cells. nt, nucleotide; pfu, plaque-forming units; T7 RNA pol., T7 RNA polymerase.

To develop a T7-based reverse genetics system, the TOSV strain H4906 was chosen, as the complete genome sequence is available in the GenBank database. H4906 was obtained by gene synthesis, and cloned into a system that relies on the T7 promoter for the synthesis of viral transcripts. The viral antigenomic full-length segments, flanked by a T7 promoter and HdV ribozyme sequence, were cloned into plasmids, as shown in Figure 1a. The plasmid pUC57 was used for the expression of the segments S and L. As the cloning of segment M into the pUC57 plasmid systematically failed, we used the vector pCC1 instead. A comparable method has been employed successfully to recover the infectious particles of the mosquito-borne phlebovirus RVFV from plasmids [27,28]. The complete system is depicted in Figure 1a,b. In all further experiments, for convenience, we refer to the wild-type (wt) virus H4906 strain as TOSV and the same virus rescued from cDNAs as rTOSV.

rTOSV was first recovered from the hamster kidney cell line BHK-21, stably expressing T7 polymerase. These cells are convenient to transfect and highly permissive to many phleboviruses. Infectious particles were detected in supernatants by plaque-forming unit assay on BHK-21 cells (Figure 1c). Co-transfection of the T7-driven plasmids encoding the full-length S, M, and L segments was essential for the rescue of TOSV, as revealed by plaque formation. Titers of rTOSV in the cell culture medium reached 10^4^ to 10^5^ plaque-forming units (pfu) per mL already, 5 days post-transfection. The peak titers remained constant over one round of amplification in BHK-21 cells (passage 1) and four consecutive passages in the monkey epithelial kidney Vero cells (passages 2 to 5), varying from 10^6^ to 10^7^ pfu/mL, which is a typical range for TOSV (Figure 1d). Together, these results demonstrate that rTOSV was rescued successfully and that titers remained stable upon passages.

### 3.2. Characterization of rTOSV Viral Particles

**Figure 2:**
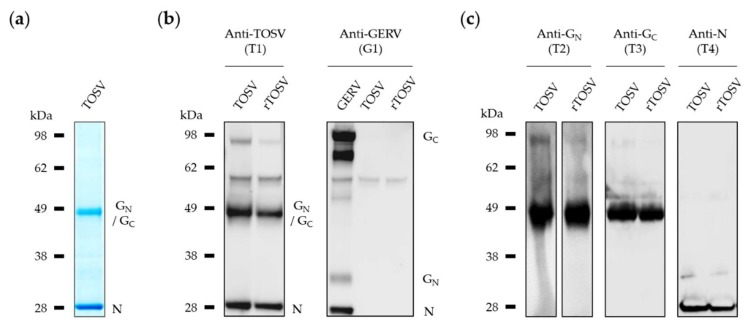
Characterization of rescued TOSV particles after amplification in BHK-21 cells and four successive passages in Vero cells. (**a**) Proteins from viral particles purified through sucrose gradient were separated by nonreducing SDS-PAGE and stained with Coomassie blue. (**b**,**c**) Viral particles of TOSV wt strain H4906, rTOSV, and Germiston virus (GERV) were analyzed by SDS-PAGE and Western blotting under non-reducing conditions. (**b**) Viral proteins were visualized using guinea pig polyclonal antisera raised against TOSV (T1) or GERV (GR1); (**c**) same as (**b**), except using either the guinea pig polyclonal antibodies T2 and T3 against TOSV G_N_ and G_C_, respectively, or the rabbit polyclonal antibody T4 against the TOSV nucleoprotein N.

We next compared rTOSV to TOSV particles. Germiston virus (GERV), a member of a different family in the *Bunyavirales* order (*Peribunyaviridae*, genus *Orthobunyavirus*) [29], was used as a control. For immunodetection purposes, polyclonal antibodies were generated in guinea pigs against purified, lysed preparations of TOSV and GERV particles (Figure S3 and Figure 2a). Antibodies were named T1 and GR1, respectively (Table 1). When viral proteins were subjected to SDS-PAGE and Western blotting using T1, the structural proteins N and envelope glycoproteins, G_N_ and G_C_, were detected in both TOSV and rTOSV virions produced from Vero cells (Figure 2b, left panel). G_N_ and G_C_ could not be resolved in the gel with this polyclonal antibody, as the two glycoproteins have similar molecular weights. A band was detected at 60 kDa, but was not specific for TOSV, as it was also found with an unrelated control serum, i.e., the serum of animals immunized with GERV protein extracts (Figure 2b, right panel). The amount of this protein in virus stocks was however marginal, as it was not even visible by Coomassie staining (Figure 2a).

To further assess the viral protein composition of virions, we generated polyclonal antibodies against each of the three major structural proteins, namely N, G_N_, and G_C_. We used soluble ectodomains of G_N_ and G_C_ produced in insect S2 cells to raise polyclonal antibodies in guinea pigs, resulting in the antisera T2 and T3, respectively (Table 1). To complete this set of tools, we generated a polyclonal antibody recognizing the TOSV nucleoprotein N, the most abundant structural protein in viral particles, by immunizing rabbits with the full-length N protein fused to a C-terminal His-tag (T4). When TOSV and rTOSV were subjected to Western blotting analysis with these antibodies, no difference was found in the composition of virions with respect to N, G_N_, and G_C_ (Figure 2c).

A band that resisted SDS dissociation was visible at about 90 kDa for both TOSV and rTOSV particles (Figure 2b,c). This band most likely corresponds to homodimers of G_N_, as no such a band could be observed with the antibody against G_C_ (T3). The newly generated glycoprotein-specific sera contributed to reveal the origin of the different proteins observed in Western blot with anti-TOSV antibodies. In addition, the electrophoretic bands of G_N_ appeared more smeared than that of G_C_ (Figure 2c), suggesting the two viral glycoproteins carry distinct glycans. Altogether, these results indicate that the composition of virions is identical, whether rescued from cDNAs or produced from cells infected with wt virus.

### 3.3. Growth Properties of the Rescued TOSV is Similar to Those of the WT Strain

**Figure 3:**
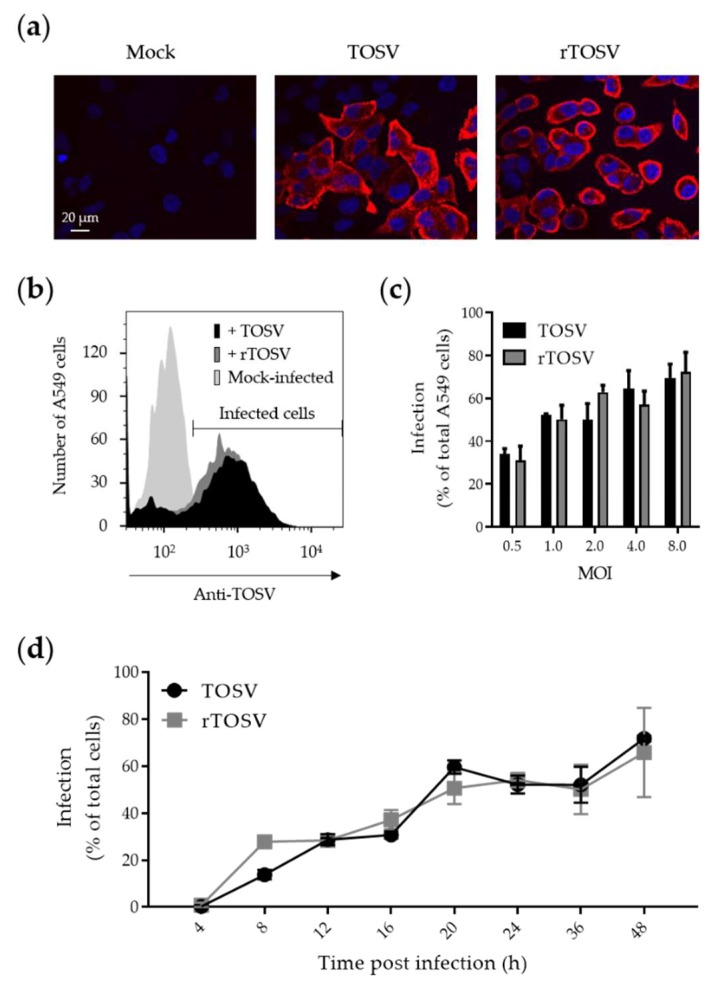
Infection of A549 cells by rTOSV and TOSV. (**a**) A549 cells were infected with TOSV and rTOSV (MOI ∼2) for 24 h and then immunostained for the intracellular TOSV structural proteins G_N_, G_C_, and N, using an anti-TOSV primary mouse polyclonal antibody and an AF568-coupled anti-mouse secondary monoclonal antibody (red). Nuclei were stained with Hoechst (blue), and samples were imaged by wide-field microscopy; (**b**) Same as in (a), except that infected cells were analyzed by flow cytometry; (**c**) A549 cells were exposed to TOSV and rTOSV at the indicated MOIs for 24 h. Infection was analyzed by flow cytometry after immunostaining against the three structural proteins G_N_, G_C_, and N. Error bars indicate SD. Simple linear regression test returned no significant difference between TOSV and rTOSV series of values; (**d**) Infection with TOSV and rTOSV (MOI ∼2) was monitored by flow cytometry in A549 cells over 48 h. Error bars indicate SD. Two-Way ANOVA with Geisser-Greenhouse correction and Sidak’s multiple comparison tests returned no significant difference between the TOSV and rTOSV series of values.

We then analyzed and compared the infection and replication of the wt TOSV strain and rTOSV in the lung epithelial A549 line as representing human cells. A549 cells were infected with both rTOSV and TOSV, and infection was analyzed 24 h later through indirect immunofluorescence of N, G_N_, and G_C_ [13] (Figure 3a). Additionally, infection was controlled by flow cytometry, which allowed for the quantitative detection of infected cells (Figure 3b). Regardless the virus strain, we found that more than 70% of A549 cells were infected at the highest MOI 24 h post-infection (Figure 3c). The fraction of infected cells increased over time and reached a plateau after 20 h, when a MOI of 2 was used (Figure 3d), showing that the signal detected corresponded to viral replication and not to the input virus. Taken together, these data indicated that the growth properties of TOSV and the rescued virus are similar.

### 3.4. Generation of a TOSV Mutant Lacking NSs Expression

**Figure 4:**
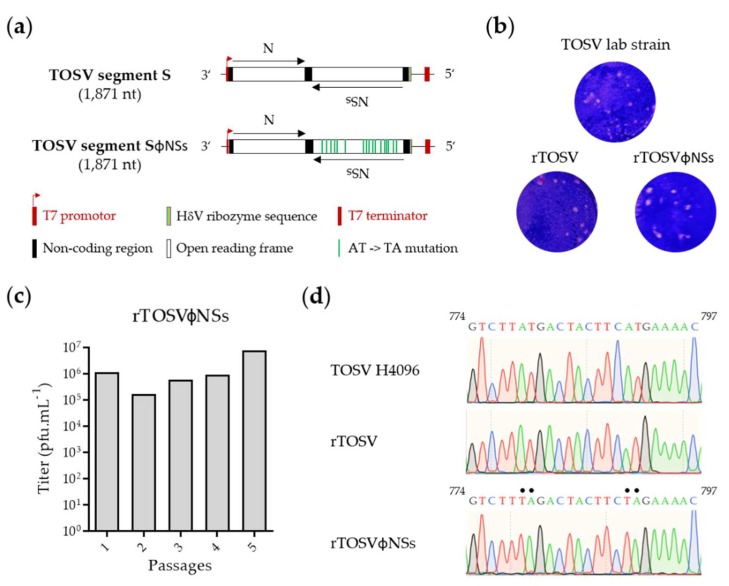
Recovery of rTOSV lacking NSs expression (rTOSVɸNSs). (**a**) Schematic representation of a rTOSV S segment encoding NSs mRNA, in which 18 AUG codons are replaced by UAG stop codons. The green bars indicate the mutations in the S segment of rTOSVɸNSs; (**b**) TOSV and rescued TOSV +/- NSs expression (rTOSV and rTOSVɸNSs, respectively) were analyzed by plaque-forming unit assay on Vero cells five days post-infection; (**c**) Titration of rTOSVɸNSs after rescue from cDNAs and amplification in BHK-21 cells (passage 1), followed by four subsequent rounds of amplification in Vero cells (passage 2 to 5); (**d**) Sequence analysis of the NSs sequence in the S segment of rTOSVɸNSs, compared to that in the S segment of rTOSV and wt TOSV carried out from vRNA purified extracts after one passage in BHK-21 cells and four subsequent passages in Vero cells. The mutations of the AUG codons at positions 779 and 790 within the NSs sequence are shown here. HdV, hepatitis d virus; pfu, plaque-forming units.

Expression of TOSV NSs from plasmid has been shown to result in downregulation of type I IFN responses [17]. To confirm that TOSV NSs indeed counteracts type I IFN responses when expressed from the viral genome, we generated a TOSV mutant lacking NSs expression (rTOSVɸNSs). Rescue of rTOSV with complete deletion of the NSs gene was not successful, despite several attempts. Additionally, swapping the NSs sequence in the S segment by that of a reporter gene such as the green fluorescent protein (GFP) was also a setback. Therefore, we chose an alternative strategy and replaced 18 AUG codons, including the authentic start codon, by UAG stop codons in the NSs nucleotide sequence (Figure 4a and Figure S2). Supernatants from transfected cells were assessed by plaque-forming unit assay using Vero cells. Plaques of the recombinant rTOSV lacking NSs expression were similar in morphology and size to those of TOSV and rTOSV (Figure 4b). The titers of rTOSVɸNSs in the supernatants of transfected BHK-21 cells varied from 10^4^ to 10^5^ pfu/mL. After one amplification in BHK-21 cells, and then four successive passages in Vero cells, titers remained stable at around 10^6^ pfu/mL (Figure 4c), slightly lower than the wt recombinant strain. The 18-point mutations in the NSs sequence were confirmed by nucleotide sequencing after the fifth round of amplification in Vero cells (Figure 4d). The sequencing of rTOSVɸNSs also confirmed that rescued viruses were derived from transfected plasmids, and not from potentially contaminating wt TOSV.

**Figure 5:**
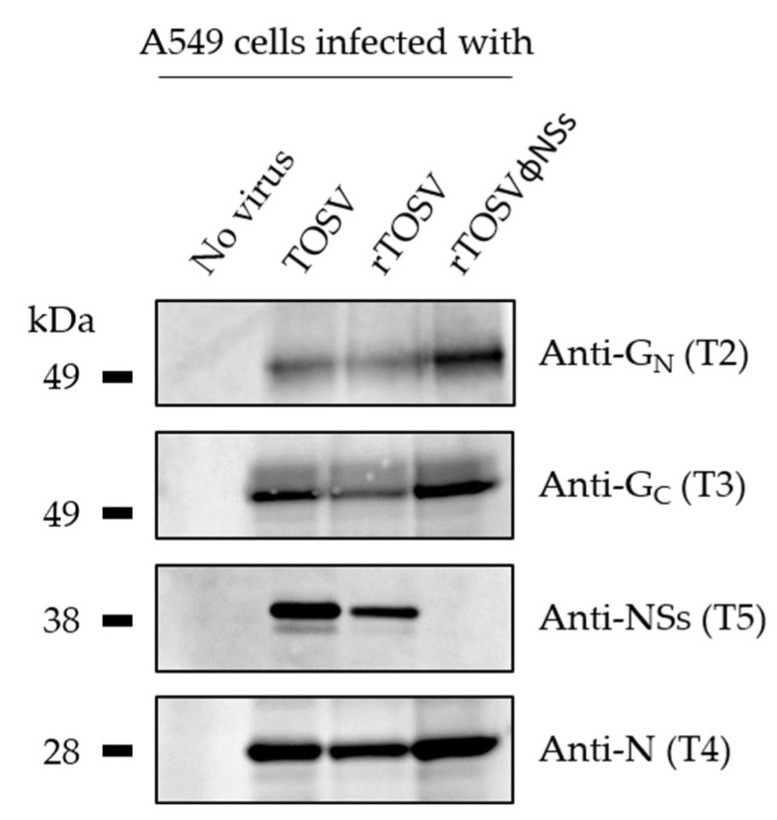
A549 cells were infected with the wt TOSV strain, rTOSV, and rTOSVɸNSs. Infected cells were lysed 24 h later, and then analyzed by SDS-PAGE and Western blotting under non-reducing conditions. The guinea pig antibodies T2 and T3 were used to detect the TOSV structural proteins G_N_ and G_C_, respectively, while the rabbit polyclonal antibodies T4 and T5 allowed the detection of the TOSV nucleoprotein N and non-structural protein NSs.

To confirm the lack of NSs expression from the rTOSVɸNSs genome, A549 cells were infected with wild-type TOSV, rTOSV, and rTOSVɸNSs at an MOI of 3 for 24 h. Infected cells were lysed and then analyzed by Western blotting with antibodies against each of the three major structural proteins and NSs. The polyclonal anti-TOSV NSs antibodies were purified from a serum of rabbits immunized with a peptide corresponding to the last 14 amino acids of the NSs C-terminal region (T5), as previously described [30]. While the structural proteins N, G_N_, and G_C_ were detected in cells infected with all the viruses, NSs was only visible in cells exposed to the wt TOSV and rTOSV (Figure 5), confirming the silencing of the NSs gene in cells infected with rTOSVɸNSs.

### 3.5. NSs Silences IFN mRNA Expression

**Figure 6:**
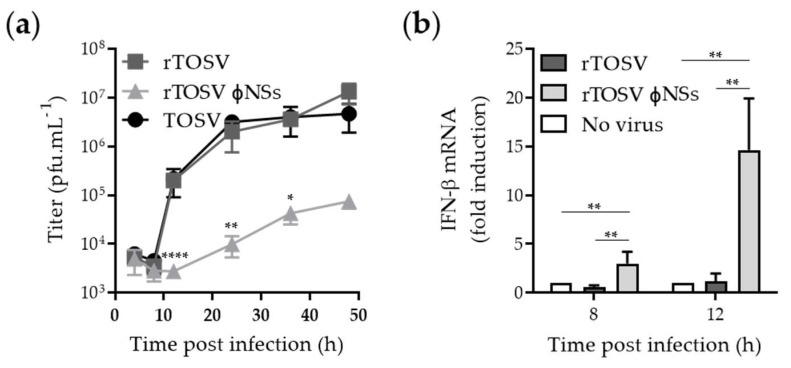
TOSV NSs counteracts IFN-b mRNA expression in A549 cells. (**a**) A549 cells were infected with TOSV, rTOSV, and rTOSVɸNSs at a MOI of 2. Supernatants from infected A549 cells were collected at indicated time points and subjected to plaque assay using BHK-21 cells. Error bars indicate SD. Two-Way ANOVA with Geisser-Greenhouse correction and Tukey’s multiple comparison tests were applied. Significant differences are indicated in the graph; (**b**) A549 cells were infected with TOSV, rTOSV, and rTOSVɸNSs (MOI ~2) for up to 12 h. Infected cells were then lyzed and total RNA was extracted and purified. IFN mRNA levels were quantified by qRT-PCR. Error bars indicate SD. Two-Way ANOVA with no correction and Sidak’s multiple comparisons tests were applied. Significant differences are indicated in the graph. *, *p* < 0.05; **, *p* < 0.01; ****, *p* < 0.0001.

In BHK-21 and Vero cells, which both have a defect in type I IFN production [31,32,33], we showed that TOSV, rTOSV, and rTOSVɸNSs replicate to similar titers (Figure 1d and Figure 4c). We next analyzed the growth properties of all three viruses in A549 cells, as this cell line is well known to be competent for both IFN production and response [34]. When A549 cells were infected with either TOSV or rTOSV, a complete cycle, from virus binding to release of infectious progeny, lasted about 12 h and reached a plateau of 10^7^ pfu/mL after 24 h (Figure 6a). The peak titer of rTOSVɸNSs was two logs lower than those of wt TOSV and rTOSV, though cells infected by these two virus strains expressed slightly lower amounts of viral proteins (Figure 5). Together, these results show that the absence of NSs expression results in a slower and weaker production of viruses in A549 cells. This further emphasizes the importance of NSs for TOSV amplification in IFN-competent cells.

To test whether NSs plays a role in TOSV escape from the type I IFN response, we infected A549 cells with rTOSV and rTOSVɸNSs. In these experiments, we used the mock-infected cells as reference. When cells were infected with rTOSVɸNSs, a strong induction of IFN-b mRNA expression was observed in comparison to cells exposed to rTOSV (Figure 6b). The difference was 20-fold at 12 h post-infection. In aggregate, these observations indicate that the TOSV NSs counteracts type I IFN-b mRNA production in infected cells.

## 4. Discussion

TOSV is a human pathogen that is re-emerging in southern Europe and northern Africa, with 250 million people exposed to the virus, and with a high seroprevalence in the Mediterranean basin—up to 50% in certain areas [2,35]. The TOSV life cycle, however, remains understudied, and the virus can be considered neglected. As a consequence, molecular tools are missing for prevention, diagnostics, and fundamental research. With this work, we report the successful recovery of infectious TOSV particles from cDNAs, as well as a panel of new antibodies against each of the major viral proteins. Using these novel tools, we were able to rescue and characterize a recombinant virus lacking expression of the non-structural protein NSs, and established the importance of the TOSV NSs protein in counteracting type I IFN production. As it stands, our work opens new perspectives for investigations into the biology of TOSV and the molecular function(s) of TOSV NSs.

RVFV was the first phlebovirus in the *Phenuiviridae* family, for which a reverse genetics system was established to rescue infectious viral particles from cDNAs [36]. Since then, similar or derivative approaches have been adapted for some other phenuiviruses, including the tick-borne Uukuniemi virus (UUKV), and the severe fever with thrombocytopenia syndrome virus (SFTSV) [37,38,39]. We have here added a T7-based reverse genetics system enabling the modification of the TOSV viral genome. Similar to approaches employed to rescue UUKV and SFTSV from cDNAs [37,39], our T7 polymerase-driven system only involves three plasmids, each coding for one of the three viral antigenomic RNA segments.

Our reverse genetics system allowed for the recovery of TOSV lacking NSs expression by introducing stop codons in the corresponding gene. Interestingly, we failed to rescue TOSV expressing GFP in place of NSs. It was also not possible to rescue TOSV lacking the full-length NSs-encoding sequence. There could be many explanations for this. The structure of the recombinant S segment might not resemble that of the authentic virus, which could compromise antigenome synthesis and genome packaging. Additionally, or alternatively, the termination sequence of the nucleoprotein N might be located in the NSs sequence, as this is the case for the phenuivirus SFTSV [37].

From earlier studies, it was not clear if TOSV counteracts type I IFN responses. The virus was shown to both suppress and induce the IFN responses in cell cultures [40,41]. The reason may be the different cell model systems used in those studies. Our data show that the presence of NSs is not required for efficient replication of TOSV in cells defective in IFN responses. In cells exhibiting robust IFN production, only the NSs-encoding viruses were able to replicate efficiently and to counteract type I IFN mRNA production. In line with our results, expression of NSs from plasmid or heterologous viral genomes suggested that TOSV NSs prevents IFN response [17,18,19]. In conclusion, the present study demonstrates that TOSV NSs, expressed in the authentic viral context, functions as a type I IFN antagonist.

In addition to TOSV, several other phleboviruses have been reported to counteract the IFN antiviral response [16,42]. Often, virulence seems to correlate with the capacity of phleboviruses to block the IFN pathway and the innate immunity in general [16,18,39,42,43,44]. The present study provides a toolbox that will lay the basis for future research, not only on the interplay between TOSV infection and innate immunity, but also on the basic molecular and cellular mechanisms governing TOSV entry, replication, assembly, and egress. Furthermore, our work opens new perspectives to develop preventive approaches, novel vaccine candidates, and specific treatments against TOSV, the prevalence of which is rapidly increasing in Europe.

## Figures and Tables

**Table 1:** List of polyclonal antibodies generated in this study.

Name	Purified	Species	Virus	Antigens	Target Protein
T1	No	Guinea pig	TOSV	Virus	N, G_N_, and G_C_
T2	No	Guinea pig	TOSV	Soluble G_N_	G_N_
T3	No	Guinea pig	TOSV	Soluble G_C_	G_C_
T4	Yes	Rabbit	TOSV	Full length N	N
T5	Yes	Rabbit	TOSV	Peptide NSs	NSs
GR1	No	Guinea pig	GERV	Virus	N, G_N_, and G_C_

**Table 2:** List of primers used for Real-time quantitative reverse transcription PCR (qRT-PCR).

Name	Sense ^1^	Sequence (5′ -> 3′)	Purpose ^2^
IFN-b-F	For.	GCCGCATTGACCATCTAT	qRT-PCR
IFN-b-R	Rev.	GTCTCATTCCAGCCAGTG	qRT-PCR
HPRT1-F	For.	CCTGGCGTCGTGATTAGTGAT	qRT-PCR
HPRT1-R	Rev.	AGACGTTCAGTCCTGTCCATAA	qRT-PCR

^1^ For., forward; Rev., reverse. ^2^ qRT-PCR, real-time quantitative reverse transcription PCR.

## Supplementary Materials

The following are available online at https://www.mdpi.com/1999-4915/12/4/400/s1, Figure S1: Sequences of the 3′ and 5′ ends of the TOSV genomic segments in the pUC57 and pCC1 plasmids, Figure S2: Alignment of the wt and mutated TOSV NSs nucleotide sequence in the S segment, and Figure S3: Sucrose-gradient purification of TOSV.
