# Supplementary material for: Novel Toscana Virus Reverse Genetics System Establishes NSs as an Antagonist of Type I Interferon Responses
Source: Viruses. 2020 Apr 4;12(4):400. doi: 10.3390/v12040400 (PMC7232479; doi:10.3390/v12040400)
Supplement: Supplementary file 1 [file viruses-12-00400-s001.pdf]

Plasmid	TOSV genomic segment end	Sequence (5’ -> 3’)
pUC57-L	T7 promotor – 3’UTR – ATG	TAATACGACTCACTATAGACACAAAGAGGCCCAAAT**ATG**
	TAA – 5’UTR – HδV ribozyme	**TAA**CAACTTTGACACTTAGAGAAACTAAGAAGGATAAAGGGTTAGGGGGGTGTTCCTTTGTTATGTCTATAAATTATTTAAGAATTGGGCGGTCTTTGTGTGGGTCGGCATGGCATCTCC
pCC1-M	T7 promotor – 3’UTR – ATG	TAATACGACTCACTATAGACACAAAGAAGGTGCTT**ATG**
	TAA – 5’UTR – HδV ribozyme	**TAA**AATAATCCATCTTCTTTCTATTATTCTCATGTATCTTTCACTCAGGGTTTAGGGGGGGAGTGGGCAGTGCATGTGTATGGCTCCGTTAGGCAGGTCTGAATCTCAGGGAAAGTATGGGAAGCAAAACAAGTTGATGGCAGTTTGGACATATTTTGTTTTGTTCTTTAAAGCACCGGTCTTTGTGTGGGTCGGCATGGCATCTCC
pUC57-S	T7 promotor – 3’UTR – ATG	TAATACGACTCACTATAGACACAGAGATTCCCGTGTATTAATCAAAAGCTATCAAC**ATG**
	TAA – 5’UTR – HδV ribozyme	**CAT**GGCTGTCTAGAAGTCTATTATTAGTTCTGGTTTAGCGATACGGGAGGTCTTTGTGTGGGTCGGCATGGCATCTCC

Figure S1. Sequences of the 3’ and 5’ ends of the TOSV genomic segments, flanked by the T7 polymerase promotor (T7 promotor) and the HδV ribozyme respectively. T7 polymerase promotor and HδV ribozyme appear in black and TOSV genome sequences in blue. The authentic ATG (or CAT) start codons and the UAA stop codons appear in blue bold. UTR, untranslated terminal region.

```
       1
NSs   ATGCAATCCAGAGCTGTCATTCTGAAGCACAAGTCTGGTTCAAGCCATAAGAGGTCTTTG 60
ϕNSs  TAGCAATCCAGAGCTGTCATTCTGAAGCACAAGTCTGGTTCAAGCCATAAGAGGTCTTTG 60

NSs   CCTAGGTTCTACATAGACTGTGACCTGGACACCTTTGATTTTGAAAAGGACTGCTCTTTA 120
ϕNSs  CCTAGGTTCTACATAGACTGTGACCTGGACACCTTTGATTTTGAAAAGGACTGCTCTTTA 120

              2                          3
NSs   ATCGAGAATGAGTTCCCTATTTACATAAAAAATTATGAGGTAGTATACAAGTCAAGACCA 180
ϕNSs  ATCGAGATAGAGTTCCCTATTTACATAAAAAATTTAGAGGTAGTATACAAGTCAAGACCA 180

                             4                               5
NSs   ACTCTTTCACACTTCCTCATAAATGGAGAATTCCCTGCAGTTCTGGGACCAGGTATGATC 240
ϕNSs  ACTCTTTCACACTTCCTCATAATAGGAGAATTCCCTGCAGTTCTGGGACCAGGTTAGATC 240

                             6
NSs   AGTGCTGTTCGAACTAGACTTTATGAGCCAACAATAAGGGAGCTTTACCAGGAATCTATT 300
ϕNSs  AGTGCTGTTCGAACTAGACTTTTAGAGCCAACAATAAGGGAGCTTTACCAGGAATCTATT 300

                                                      7
NSs   CATCAGCTAAAAAGGAGCAATAAGAAATATCTCTTGTCTGCCCTCAGATGGCCAACAGGA 360
ϕNSs  CATCAGCTAAAAAGGAGCAATAAGAAATATCTCTTGTCTGCCCTCAGTAGGCCAACAGGA 360

NSs   ATCCCCTCTCTTGAGTTCATAGATTACTATTTCGAGGAGCTCCTGTTCTTGTCCGAGTTT 420
ϕNSs  ATCCCCTCTCTTGAGTTCATAGATTACTATTTCGAGGAGCTCCTGTTCTTGTCCGAGTTT 420

NSs   GATCCAGGATCTATTCAGAGATATCTCAAATTACTGGTCAAGGCTTCTGGACTCTACTGT 480
ϕNSs  GATCCAGGATCTATTCAGAGATATCTCAAATTACTGGTCAAGGCTTCTGGACTCTACTGT 480

NSs   TCAACCATTGAGGAGCAAATAGTGGAAATTCATAGGAGGGTTCTCCTAGAAGGCAAGAAG 540
ϕNSs  TCAACCATTGAGGAGCAAATAGTGGAAATTCATAGGAGGGTTCTCCTAGAAGGCAAGAAG 540

        8                             9                  10
NSs   CATGGATTAACTGCTTTTGACCTCCCAGGAAATGACATCCTCGGAGACATATGTGTGGTT 600
ϕNSs  CTAGGATTAACTGCTTTTGACCTCCCAGGAATAGACATCCTCGGAGACATTAGTGTGGTT 600

                                                   11
NSs   CAAGCAGCACGGGTGACTAAATTGGTTGCAAAGGCATTTTCTAGAATGACCAGAGACTCC 660
ϕNSs  CAAGCAGCACGGGTGACTAAATTGGTTGCAAAGGCATTTTCTAGATAGACCAGAGACTCC 660

            12
NSs   CAACTGATGATATACTTTTCAATAAGCCCAGTGGAATTAGTTCTGAGCAAGCTTGATAGG 720
ϕNSs  CAACTGTAGATATACTTTTCAATAAGCCCAGTGGAATTAGTTCTGAGCAAGCTTGATAGG 720

          13                                 14                  15
NSs   AAAGATGACAAGAAGGCTAAGGCAAAGGGTTTACTATCAATGAGTGCTGCCAAGTCTTAT 780
ϕNSs  AAAGTAGACAAGAAGGCTAAGGCAAAGGGTTTACTATCATAGAGTGCTGCCAAGTCTTTA 780

               16
NSs   GACTACTTCATGAAAACAGACCTGAGATTTAGAGAAACTGCTCTTTCCACCTTCTGGGCT 840
ϕNSs  GACTACTTCTAGAAAACAGACCTGAGATTTAGAGAAACTGCTCTTTCCACCTTCTGGGCT 840

                         17                       18
NSs   AAAGATTGGCCCACAATACATGAGTCTGTGCTGTCTAATAGACGATGCCCTAAGGAAGAC 900
ϕNSs  AAAGATTGGCCCACAATACTAGAGTCTGTGCTGTCTAATAGACGTAGCCCTAAGGAAGAC 900

NSs   AAGAGAGTGACAAAGTGGCTGCCTAGTCCCCCCCACTACCCACCCCTATGA 951
ϕNSs  AAGAGAGTGACAAAGTGGCTGCCTAGTCCCCCCCACTACCCACCCCTATGA 951
```

Figure S2. Nucleotide sequence alignment of TOSV NSs (H4906) strain and the derived mutant ϕNSs, the 18 first ATG codons of which were replaced by TAG stop codons. The blue numbers indicate the ATG codons that have been mutated in the ϕNSs sequence. ATG codons appear in bold red in the wild type NSs sequence when they have been replaced by TAG stop codons, which appear in bold green in the ϕNSs sequence.

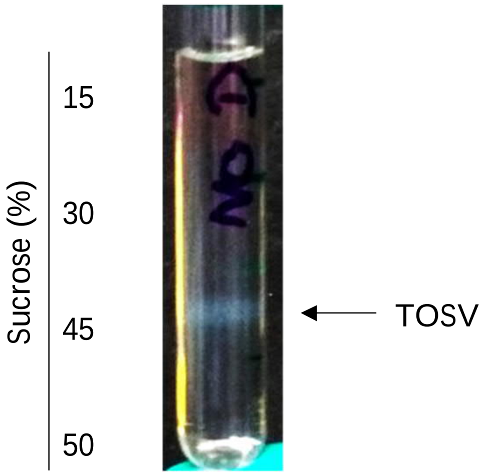

Figure S3. The picture shows a sucrose linear gradient after ultracentrifugation with a large white band that corresponds to TOSV.
